# Effects of Prior Heat Treatment and Induction Hardening on the Properties of JIS SUJ3 Bearing Steel

**DOI:** 10.3390/ma18081797

**Published:** 2025-04-15

**Authors:** Shao-Quan Lu, Liu-Ho Chiu, Pei-Jung Chang, Chung-Kwei Lin

**Affiliations:** 1 Department of Mechanical and Materials Engineering, Tatung University, Taipei 104327, Taiwan; d11101001@o365.ttu.edu.tw; 2 Research Center of Digital Oral Science and Technology, College of Oral Medicine, Taipei Medical University, Taipei 110301, Taiwan; peronchang@tmu.edu.tw; 3 Graduate Institute of Manufacturing Technology, National Taipei University of Technology, Taipei 106344, Taiwan; 4 School of Dental Technology, College of Oral Medicine, Taipei Medical University, Taipei 110301, Taiwan

**Keywords:** bearing steel, prior heat treatment, induction hardening, microhardness, retained austenite, residual stress, wear

## Abstract

Bearing steels are frequently used in highly loaded components, such as roller bearings, due to their excellent hardenability and wear resistance. Microstructure, hardness, and residual stress distribution of the bearings significantly affect the wear resistance of the parts. In the present study, experiments investigated the effects of austenitizing temperature (850, 900, and 950 °C), with or without cryogenic treatment, and induction hardening treatment (9 and 12 kW) on the microstructure, microhardness, the amount of retained austenite, surface residual stress, and wear behavior of JIS SUJ3 steel. The experimental results revealed that the austenitized specimens’ microstructure consisted of martensite, retained austenite, and dispersed granular alloy carbide exhibiting high hardness. After cryogenic or induction hardening treatment, the surface residual stress of austenitized specimens exhibited compressive stress rather than its original tensile stress state. The induction hardening treatment can significantly increase the microhardness of austenitized specimens, followed by quenching. Furthermore, the induction-hardened surface possessed less retained austenite. For practical industrial applications, a prior austenitizing heat treatment at 950 °C followed by hardening with an induction power of 12 kW was the optimal parameter for JIS SUJ3 bearing steel. The maximum microhardness and surface residual stress were 920 HV_0.3_ and −1083 MPa, respectively, while the lowest weight loss was 0.5 mg after the 10,000-revolution wear test.

## 1. Introduction

With the rapid development of industrial machinery, the requirements for wear resistance and fatigue resistance of rolling parts such as bearing rings, ball screws, and other mechanical components have increased to ensure their proper function, and the selection of materials is a crucial issue. The most commonly used material in manufacturing precision and motion components is high chromium bearing steel [1]. The damages inflicted on the bearing steel are mainly due to wear and fatigue caused by the conditions of their usage. Thus, to ensure superior comprehensive mechanical properties and high dimensional stability, the bearing steel should exhibit high hardness and good wear resistance, which directly impact its lifespan. JIS SUJ3 steel is a high-quality alloy steel that has excellent wear resistance after hardening treatment, with a hardness of over 700 HV and a tensile strength of 1200 MPa.

Precision and motion parts made of bearing steel are usually required with good wear resistance to ensure their proper function. A major factor that affects wear behaviors is the hardness of the materials used to manufacture these mechanical parts. Other factors that affect resistance to mechanical damage include heat treatment procedures. The heat treatment of bearing steel is often conducted by quenching and tempering. The bearing steel, however, often forms retained austenite after quenching. This high content of retained austenite is due to the high carbon content in the bearing steel, as reported by Rivero et al. [2] and Roy et al. [3,4]. The transformation of the retained austenite into martensite can occur when external environmental factors such as temperature and load are introduced. This can negatively impact the stability of precision and motion parts in their usage [5,6,7].

High-temperature tempering, cryogenic treatment, and shot peening treatment are typical methods for alleviating the above-mentioned concerns about retained austenite. High-temperature tempering treatment causes a significant decrease in hardness, while shot peening treatment can only eliminate the retained austenite up to a limited depth in the subsurface region [8,9,10,11], and the resulting surface roughness does not meet the requirement of a bearing part. Unlike the other two methods, cryogenic treatment further induces phase transformation of the retained austenite through heat exchange generated at low temperatures, as reported by Bensely et al. [12], Senthilkumar et al. [13] and Mahdi et al. [14]. Its advantages not only include reducing the amount of retained austenite but also significantly relaxing residual stress [15,16]. Thus, cryogenic treatment is a preferable method. However, the cost and production time of cryogenic treatment are relatively high due to its technical threshold.

To reduce production time and costs to further reduce the consumption of energy, induction hardening is a good choice to consider. Shorter processing times make induction hardening treatment more affordable. Furthermore, induction hardening treatment also significantly improves surface properties, especially surface residual stress. Hu et al. [17], Gao et al. [18], and Hayama et al. [19] investigated the surface properties after induction quenching, and their results revealed that induction hardening treatment creates compressive residual stress and prolonged service life when compared to those mechanical parts with a tensile stress surface [20,21,22]. Moreover, the parts treated by induction hardening respond to less grain growth, possibly higher hardness, and a lower amount of retained austenite due to its rapid cooling rate.

Numerous studies explored the relationship between the heat treatment (quenched, tempered, and cryogenic treatment) and the resulting properties (hardness and the retained austenite content) and the wear behavior of bearing steel. The effects of prior treatment and induction hardening treatment on the dry wear behavior of bearing steel were not investigated in detail. The aim of the present study is to investigate the influence of different parameters of prior heat treatment (austenitization and cryogenic treatment) on the properties of induction-hardened JIS SUJ3 steel. In this study, JIS SUJ3 steel was selected for investigation. Firstly, the specimens were austenitized at various temperatures (850, 900, and 950 °C), quenched, processed with or without cryogenic treatment, and tempered to achieve a hardness of 62 ± 1 HRC to meet the requirements for industrial use in precision and motion parts. Subsequently, induction hardening with a power of 9 and 12 kW was applied to the prior heat-treated specimens. The microhardness, the amount of retained austenite, and the surface residual stress of the specimens were examined to evaluate the effects of different treatment parameters. In addition, block-on-roller type dry wear testing was used to compare the wear resistance. The properties and wear behavior of JIS SUJ3 steel specimens with different treatments were investigated for practical industrial applications.

## 2. Materials and Methods

### 2.1. Heat Treatment of Specimens

The specimens used in the experiment were made of JIS SUJ3 steel, a bearing steel manufactured by Gloria Material Technology Corporation (Tainan, Taiwan). The steel bar was spheroidized and machined to form cubic specimens with a dimension of 12.7 × 12.7 × 12.7 mm with chemical compositions listed in Table 1. The size of the specimens was designed to fix the specimen with a sample holder for wear testing according to the ASTM G77-17 specification [23].

The machined specimens were then heat-treated in a salt bath using three different austenitization temperatures. First, the specimens were preheated at a heating rate of 50 °C/min to 600 °C. Then, the specimens were austenitized at 850, 900, and 950 °C for 30 min and quenched to 100 °C with oil. After quenching, some specimens were immersed in liquid nitrogen at −196 °C for 2 h (cryogenic treatment). At the final stage, the quenched and the cryogenic-treated specimens were tempered at 200 °C for 1 h and cooled with water. Schematic illustrations of the temperature-time diagrams for QT and QCT treatments are shown in Figures S1a and S1b, respectively.

The effects of the austenitizing and cryogenic treatments were confirmed by examining surface hardness. The specimens, subjected to various conditions, exhibited a surface hardness of 62 ± 1 HRC, as determined using a Rockwell hardness tester (FUTURE-TECH FR-3e, Kawasaki, Japan) with an applied load of 150 kg.

### 2.2. Induction Hardening of Austenitized Specimens

The austenitized SUJ3 specimens immediately underwent induction hardening treatment. A digital controllable induction hardening system consists of an inductor coil, an induction power supply, and a circulating cooling water system. It is also equipped with a controllable mobile stage. The inductor was made of copper and applied with a frequency of 200 kHz. After austenitization, induction power levels of 9 and 12 kW were immediately applied, and specimens were quenched by moving them at a rate of 2.5 mm/s. After the induction hardening treatment, specimens were tempered again at 200 °C for 1 h. Table 2 summarizes all the parameters of austenitizing, cryogenic and induction hardening treatment, and the respective specimen codes. Three specimens for each sample code were prepared to ensure the reproducibility and stability of the experiments.

### 2.3. Characterizations of Austenitized and Induction-Hardened Specimens

After the austenitizing, cryogenic, and induction hardening treatments, the specimens were investigated to reveal the microhardness, the percentage of retained austenite, and the surface residual stress.

#### 2.3.1. Microhardness

The microhardness (HV) values as a function of depth (from surface to 6 mm) were determined by a Matsuzawa Vickers hardness tester (MXT50, Akita, Japan) to reveal the effects of various process parameters. Three specimens for each sample code (*n* = 3) were prepared and tested five times using a load of 300 g (2.94 N) and holding for 10 s. The microhardness (mean ± standard deviation) in the middle of the three tested specimens was used for comparison to reveal the effects of austenitizing, cryogenic, and induction hardening treatments with different parameters.

#### 2.3.2. X-Ray Diffraction

A μ-X360s portable X-ray diffractometer (Pulstec Industrial Co., Ltd., Shizuoka, Japan) was used to determine the retained austenite content and surface residual stress using the two-dimensional cos*α* method [24,25,26]. A Cr target operating at 30 kV and 1.5 mA was used for X-ray diffraction with a diffraction angle set at 0° and 35° to determine the percentage of retained austenite and residual stress, respectively. The X-ray diffraction parameters are summarized in Table 3. The amount of retained austenite and residual stress was performed at three different locations for each treated specimen. Before the experiments, the X-ray diffractometer was aligned and validated using a stress-free iron powder sample for calibration. Equation (1) was used to calculate the retained austenite content (γ%) [27]. In the equation, I and R are the integrated intensity and theoretical relative intensity, respectively. The Greek letters γ and α indicate austenite and ferrite, respectively.

γ% = (Iγ/Rγ)/[(Iγ/Rγ) + (Iα/Rα)] × 100%(1)

### 2.4. Dry Wear Behavior of Austenitized and Induction-Hardened Specimens

To investigate the effect of different parameters on the wear behavior of the specimens, a multi-functional abrasion tester (PLINT TE53, Phoenix Tribology Ltd., Kingsclere, Berkshire, UK) was used to conduct the dry wear test. A schematic illustration of the experimental setup is shown in Figure S2a. The wear tester is a computer-controlled block-on-roller contact instrument in which a cubic block (specimen) is in contact with a roller, as shown in Figure S2b. In the case of the wear test, the specimen experiences one contact cycle per revolution at a constant contact normal load (92 N) with a roller rotation speed of 250 rpm. A SUJ2 steel roller with a diameter of 60 mm and a hardness of 62 ± 1 HRC was used for the wear test that was performed under dry friction conditions with a controlled temperature of 25 ± 1 °C and a relative humidity of 70 ± 5%. The test sample was ultrasonically cleaned with alcohol, and the weight loss was measured using a microbalance every 2000 revolutions. Each sample experienced a total revolution of 10,000 cycles for the dry wear test. All tests were repeated three times. After the wear test, the surface morphology of the worn specimen was examined by field emission scanning electron microscopy (Hitachi SU8000, Tokyo, Japan).

## 3. Results and Discussion

### 3.1. Heat Treatment of the JIS SUJ3 Specimen

#### 3.1.1. The Effect of Austenitizing Temperatures on the JIS SUJ3 Specimen

**Figure 1:**
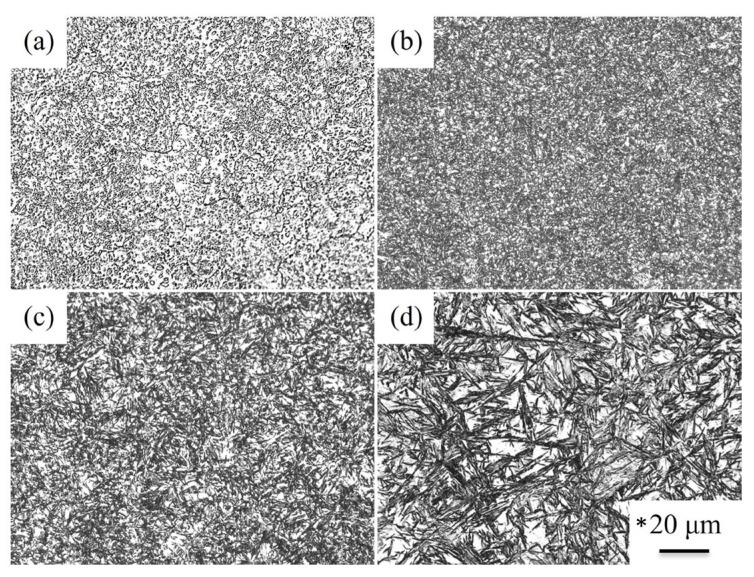
Microstructure of the (**a**) as-received SUJ3 specimen, (**b**) 850 QT, (**c**) 900 QT, and (**d**) 950 QT specimens. * The scale bars were the same for these OM images.

Figure 1 shows the microstructure of the specimen after the austenitization treatment (at 850, 900, and 950 °C) and quenching to 100 °C. The microstructure of the as-received SUJ3 specimen was shown in Figure 1a, where the spheroidal Fe_3_C cementite particles were uniformly distributed within the ferrite matrix. Figure 1b–d show the microstructure of the SUJ3 specimen after the austenitization treatment at 850, 900, and 950 °C, respectively. The ferrite matrix transformed into a martensite matrix can be observed after quenching. The proportion of the cementite decreased with increasing austenitizing temperature. However, the amount of the retained austenite increased with increasing austenitizing temperature. This is due to the higher austenitizing temperature increasing the dissolution ratio of the cementite. Moreover, high carbon content causes the martensitic transformation temperature (Ms) to decrease, thereby inducing an increase in the volume of the retained austenite. A higher magnification of these SEM images is available in Figure S3a–d for more detailed investigations.

**Figure 2:**
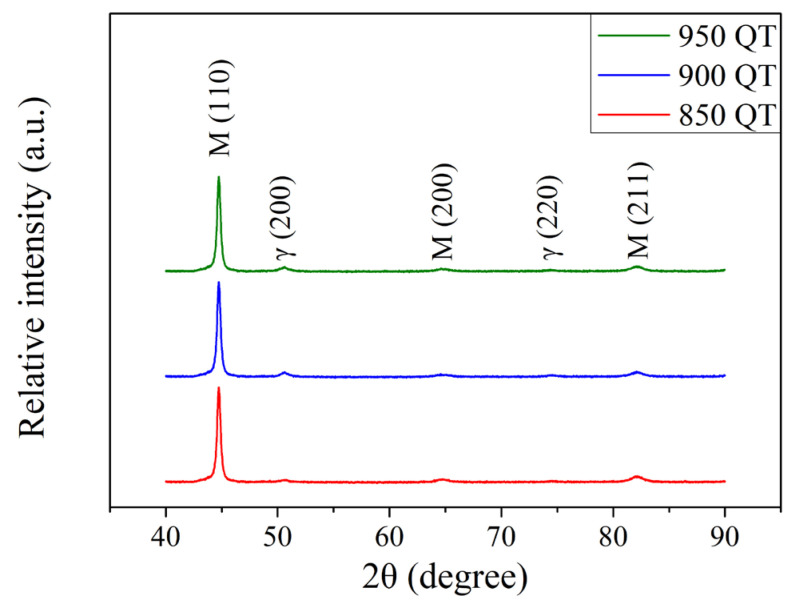
XRD patterns of SUJ3 specimens after austenitizing at various temperatures.

After austenitizing and quenching treatments, a Vickers hardness tester was used to check the hardness of the SUJ3 specimen. The hardness of the specimen was 715 ± 5, 725 ± 8 and 728 ± 15 HV_0.3_ after austenitization at 850, 900, and 950 °C, respectively. A slight increase in microhardness with increasing austenitizing temperature was observed but without significant difference. To further examine the condition of the amount of retained austenite, a portable X-ray diffractometer was used, and the measuring principle of the single incident angle method (cosα method) was applied [24,25,26]. Figure 2 shows the resulting X-ray diffraction patterns. The SUJ3 specimen, after undergoing austenitizing and quenching treatments, exhibited a distinct martensitic phase (PDF No. 44-1290) and a couple of very weak retained austenite diffraction peaks (PDF No. 65-4150). More detailed information concerning these two powder diffraction files (PDF No. 44-1290 and PDF No. 65-4150) is available in Tables S1 and S2. The peak intensities of austenite increased with increasing austenitizing temperature. The amount of retained austenite of the specimen austenitized at 850 °C was 3.4 ± 0.3%. By increasing the temperature to 900 °C, the amount of austenite retained increased to 10.4 ± 0.7% and further increased to 15.5 ± 0.6% at 950 °C. It should be pointed out that while carbon content can significantly affect the Ms temperature, the amount of retained austenite increased due to the cementite dissolved in the lattice (carbon concentration of martensite).

In addition to the retained austenite, the martensitic transformation was accompanied by volume expansion, and the boundary effect resulted in a tensile surface residual stress. The residual stress on the surface of the specimen austenitized at 850 °C was 53 ± 12 MPa. It increased gradually to 78 ± 17 and 115 ± 15 MPa at an austenitizing temperature of 900 and 950 °C, respectively. Table 4 summarizes the microhardness, the retained austenite content, and the surface residual stress of the SUJ3 specimen austenitized at various temperatures.

#### 3.1.2. The Effect of Cryogenic Treatment on the JIS SUJ3 Specimen

**Figure 3:**
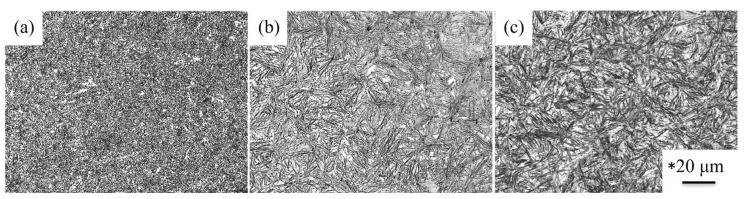
Microstructure of (**a**) 850 QCT, (**b**) 900 QCT, and (**c**) 950 QCT specimens. * The scale bars are the same for these OM images.

In industrial applications, the cryogenic treatment is commonly used to achieve dimensional stabilization after quenching. Figure 3 shows the microstructure of the austenitized specimens followed by cryogenic treatment. Figure 3a–c show the microstructure after the cryogenic treatment of the SUJ3 specimen with austenitizing temperatures of 850, 900, and 950 °C, respectively. A major difference observed was the proportion of the retained austenite, which was smaller than those in Figure 1b–d. It should be mentioned that martensitic transformation of the retained austenite occurred due to the heat exchange [8,9,10,11] and resulted in a decrease in the percentage of retained austenite after cryogenic treatment. However, the cryogenic treatment had no obvious effect on the microhardness of the SUJ3 specimen. After the cryogenic treatment, the microhardness of the specimen was 718 ± 3, 723 ± 5, and 730 ± 8 HV_0.3_ with austenitizing temperatures of 850, 900, and 950 °C, respectively. These SEM images are magnified in Figure S4a–c for better observations.

**Figure 4:**
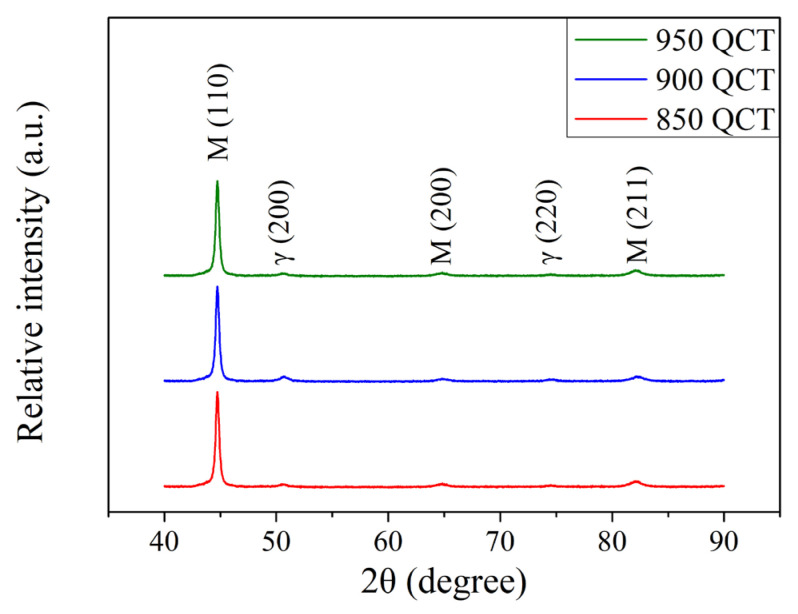
XRD patterns of the SUJ3 specimen after austenitizing at different temperatures and cryogenic treatment.

The XRD patterns of the SUJ3 specimen after cryogenic treatment at various austenitizing temperatures are shown in Figure 4. The peak intensities of austenite decreased after the cryogenic treatment. A similar observation was reported by Chen et al. [6] and Pöhl et al. [7]. The amount of retained austenite of the specimen decreases to 3.5 ± 0.2, 5.2 ± 0.3 and 7.4 ± 0.3% after cryogenic treatment for samples austenitized at 850, 900, and 950 °C, respectively. Moreover, the residual stress on the surface of the specimen changed from a tensile stress to a compressive stress of −61 ± 11, −92 ± 8 and −132 ± 14 MPa after cryogenic treatment for 850, 900, and 950 °C austenitized samples, respectively. Bensely et al. [12] and Senthilkumar et al. [13] reported similar phenomena. The cryogenic treatment is accompanied by volume contraction. Therefore, the surface residual stress appears as compressive residual stress. Table 5 summarizes the important results of the SUJ3 specimens austenitized at 850, 900, and 950 °C and subsequently underwent a cryogenic treatment.

### 3.2. Induction Hardening of the JIS SUJ3 Specimen

#### 3.2.1. The Effect of 9 kW Induction Hardening Treatment on the JIS SUJ3 Specimen

**Figure 5:**
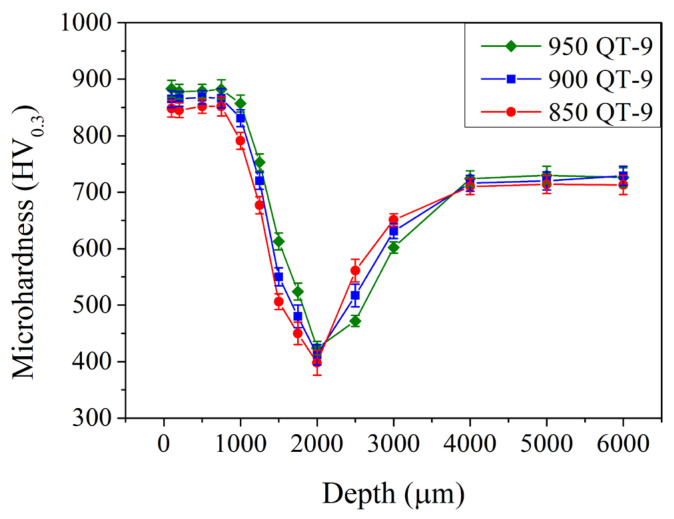
Microhardness profiles of specimens after various austenitizations followed by 9 kW induction hardening.

In Section 3.1, the effects of austenitizing temperature and cryogenic treatment were investigated. The higher the austenitizing temperature, the higher the amount of retained austenite and surface residual tensile stress. Cryogenic treatment reduces the amount of retained austenite and converts the residual stress into a compressive state. Different austenitizing temperatures and cryogenic treatment were examined, and the effects of induction hardening treatment were investigated further. The mechanical properties of the induction-hardened specimen as a function of depth were analyzed using the Vickers microhardness test with an applied load of 300 g. Figure 5 shows the microhardness in the subsurface region of the induction-hardened specimen with different prior austenitizing temperatures. After the induction hardening treatment, the microhardness increased significantly at the subsurface region up to a relatively deep depth of 0.8 mm. The microhardness exhibited a maximum of 845 ± 11, 865 ± 8, and 890 ± 12 HV_0.3_ with a prior austenitizing temperature of 850, 900, and 950 °C, respectively. It decreased continuously to a minimum of 400 ± 9, 410 ± 6, and 420 ± 6 HV_0.3_ at a depth of 2.0 mm. Then, it increased gradually to 725 ± 20 HV_0.3_ at depths of 4.0 mm and deeper. A similar microhardness profile was also observed in the literature [17,18,19]. The hardened layer (up to 0.8 mm) was attributed to the induction-hardened treatment that included the heat transfer, temperature gradient, and proportion of cementite dissolved in the lattice (carbon concentration of martensite) due to the rapid heating rate and cooling rate.

**Figure 6:**
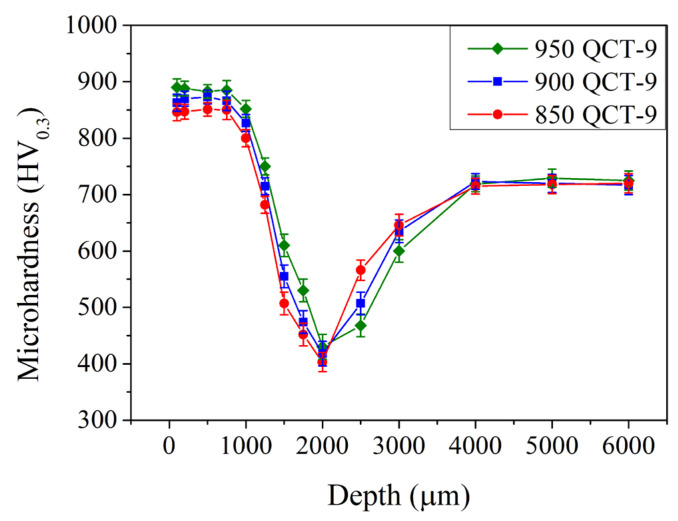
Microhardness profiles of specimens after various austenitizations followed by cryogenic treatment that were induction-hardened by 9 kW.

Figure 6 shows the microhardness in the subsurface region of the induction-hardened specimen with different prior austenitizing temperatures and cryogenic treatment. Compared to those without cryogenic treatment (Figure 5, results of QT-9 samples), specimens with cryogenic treatment exhibited a similar microhardness of 840–890 HV_0.3_ up to a depth of 0.8 mm. The microhardness decreased continuously until reaching a minimum at a depth of 2.0 mm when treated with a prior cryogenic treatment. Thereafter, it increased gradually to 725 ± 20 HV_0.3_ at depths of 4.0 mm and deeper. Thus, the cryogenic treatment does not significantly increase or decrease the microhardness in the subsurface region. However, raising the prior austenitizing temperature (up to 950 °C) increased the maximum microhardness and affected the depth of hardening in the induction-hardened specimen.

**Figure 7:**
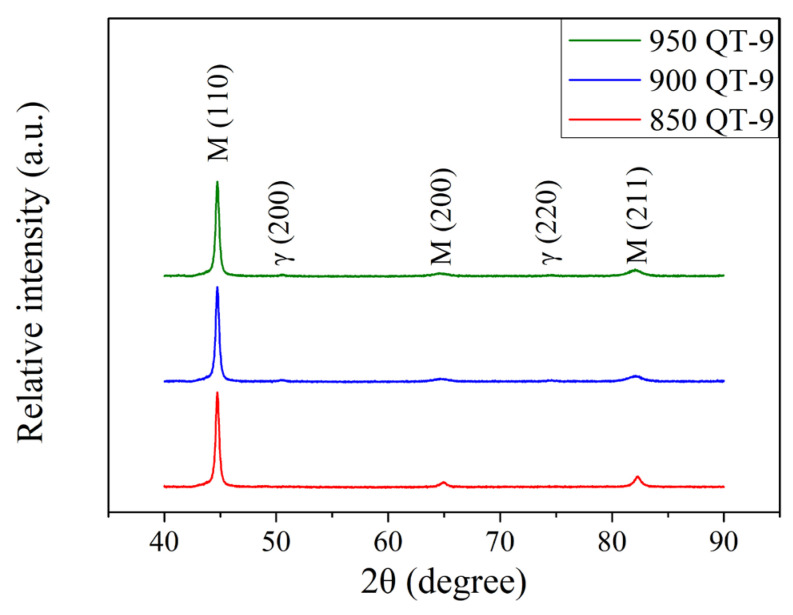
XRD patterns of specimens after various austenitizations followed by 9 kW induction hardening.

**Figure 8:**
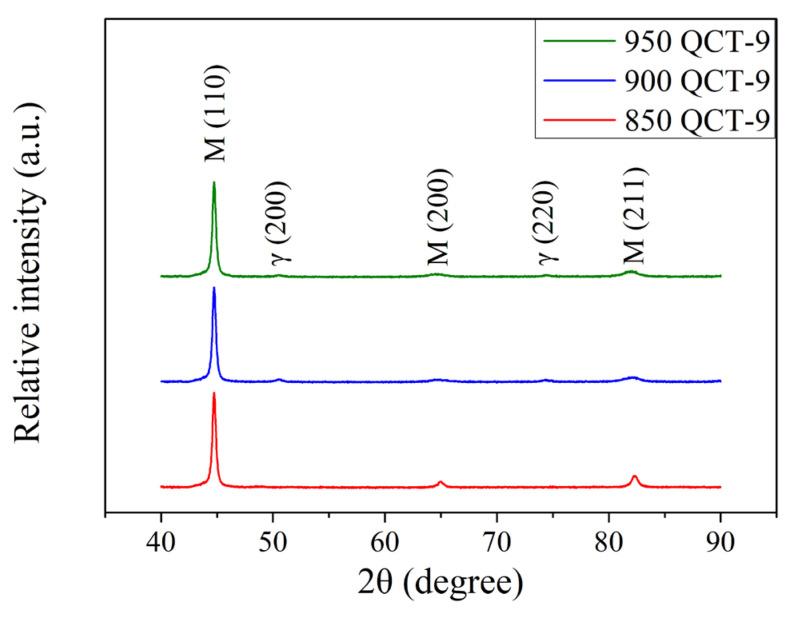
XRD patterns of specimens after various austenitizations followed by cryogenic treatment that were induction-hardened by 9 kW.

The XRD patterns of induction-hardened specimen with an induction power of 9 kW and the different prior austenitizing temperatures are shown in Figure 7. Compared to specimens without an induction hardening treatment (Figure 2), samples that underwent induction hardening treatment exhibited lower the peak intensities of austenite. It can be noted that the rapid cooling rate of induction hardening treatment is due to water sprayed during the process. The faster the quenching treatment cooling rate, the lower the amount of retained austenite. This shows a similar trend as reported by Babasafari et al. [28] and Cao et al. [29]. Figure 8 shows the XRD patterns of the induction-hardened specimen with different prior austenitizing temperatures and cryogenic treatment. Similar to the results of Figure 7 (QT-9 samples), the peak intensities of austenite remained at a relatively low level. The amount of retained austenite of induction-hardened specimens without prior cryogenic treatment was 5.3 ± 0.7, 7.3 ± 0.5 and 9.4 ± 0.9% at austenitizing temperatures of 850, 900 and 950 °C, respectively. The corresponding residual stress on the surface of the specimen was −951 ± 13, −977 ± 17 and −974 ± 9 MPa. The amount of retained austenite in the induction-hardened specimens with prior cryogenic treatment was 4.9 ± 0.4, 6.5 ± 0.6 and 8.7 ± 0.7% at austenitizing temperatures of 850, 900 and 950 °C, respectively. The corresponding residual stress on the surface of the specimen was −900 ± 20, −922 ± 15 and −915 ± 19 MPa. These results indicate that prior cryogenic treatment does not significantly affect the amount of retained austenite and residual stress after induction hardening treatment. Table 6 summarizes the amount of retained austenite and surface residual stress of the SUJ3 specimens austenitized at 850, 900, and 950 °C and followed by a 9 kW induction hardening treatment.

#### 3.2.2. The Effect of 12 kW Induction Hardening Treatment on the JIS SUJ3 Specimen

**Figure 9:**
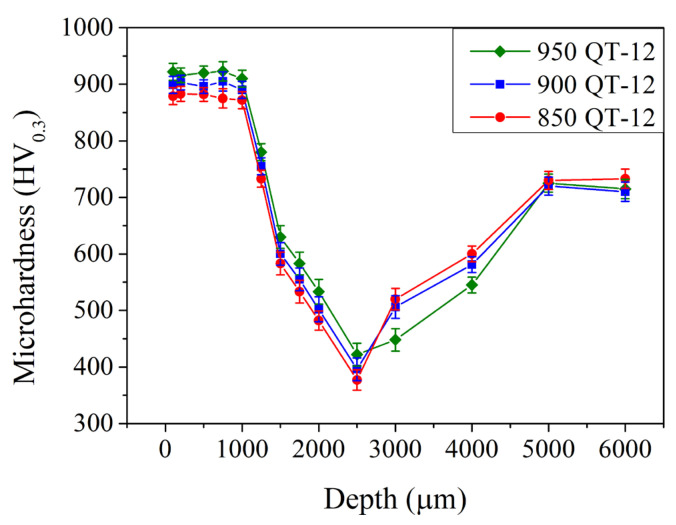
Microhardness profiles of specimens after various austenitizations followed by 12 kW induction hardening.

Figure 9 shows the microhardness in the subsurface region of the induction-hardened specimen with different prior austenitizing temperatures. After induction hardening, the microhardness increased significantly at the subsurface region up to a relatively deep depth of 1.0 mm. The microhardness exhibited a maximum at a depth of 0.1–1.0 mm and was 885 ± 6, 900 ± 5, and 920 ± 10 HV_0.3_ with prior austenitizing temperatures of 850, 900, and 950 °C, respectively. It decreased continuously to a minimum of 380 ± 12, 400 ± 9, and 420 ± 8 HV_0.3_ at a depth of 2.5 mm. Then, it increased gradually to 725 ± 20 HV_0.3_ at depths of 5.0 mm and deeper. As reported by Hu et al. [17] and Gao et al. [18], increasing the power of induction-hardening treatment increases microhardness in the induction-hardened region. In the present study, increasing the power of induction hardening treatment (up to 12 kW) increased the microhardness and the affected depth of the specimen.

**Figure 10:**
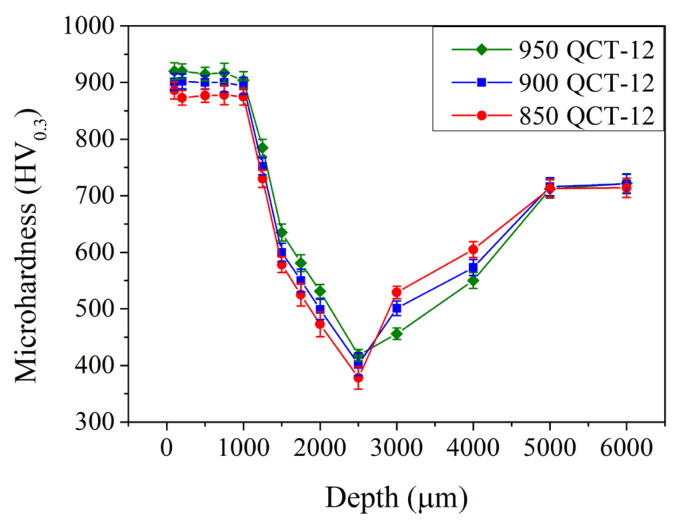
Microhardness profiles of specimens after various austenitizations followed by cryogenic treatment that were induction-hardened by 12 kW.

Figure 10 shows the microhardness in the subsurface region of the induction-hardened specimens with various prior austenitization and cryogenic treatments. Compared to those without a prior cryogenic treatment (Figure 9, QT-12 samples), the microhardness remained 880–930 HV_0.3_ up to a depth of 1.0 mm and decreased continuously to a minimum microhardness at a depth of 2.5 mm when treated with a prior cryogenic treatment. Thereafter, it increased gradually to 725 ± 20 HV_0.3_ at depths of 5.0 mm and further. The prior cryogenic treatment does not significantly increase or decrease the microhardness in the subsurface region.

**Figure 11:**
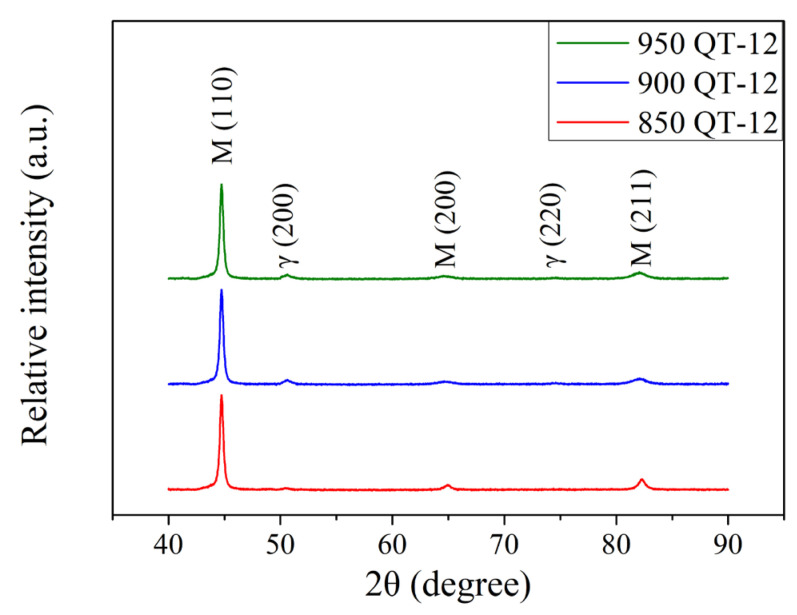
XRD patterns of specimens after various austenitizations followed by 12 kW induction hardening.

**Figure 12:**
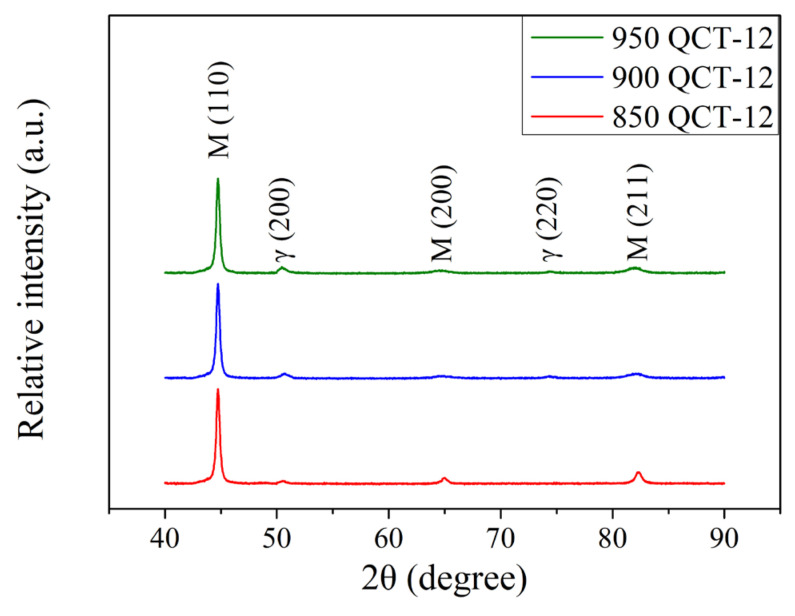
XRD patterns of specimens after various austenitizations followed by cryogenic treatment that were induction-hardened by 12 kW.

The XRD patterns of the 12 kW induction-hardened specimen with different prior austenitizing temperatures are shown in Figure 11. After 12 kW induction hardening, there was an increase in the peak intensities of austenite when compared to specimens treated at a power of 9 kW (Figure 7). This phenomenon is similar to those reported by Kaiser et al. [30] and Fisk et al. [31]. It should be pointed out that the increase in the power resulted in the specimen reaching a higher austenitizing temperature during the induction hardening treatment. Figure 12 shows the XRD patterns of 12 kW induction-hardened specimens with different prior austenitizing temperatures and cryogenic treatment. The peak intensities of austenite remained at a relatively low level. Compared to those without the prior cryogenic treatment (Figure 11, QT-12 samples), the effect of cryogenic treatment on induction-hardened specimens is not significant. The amount of retained austenite of the induction-hardened specimen without prior cryogenic treatment was 6.2 ± 0.3, 8.9 ± 0.4, and 11.8 ± 0.3% at austenitizing temperatures of 850, 900, and 950 °C, respectively. The corresponding surface residual stress of the specimen was −1068 ± 3, −1073 ± 11, and −1083 ± 5 MPa. The amount of retained austenite of the induction-hardened specimen with prior cryogenic treatment was 5.8 ± 0.2, 8.5 ± 0.4, and 11.3 ± 0.4% at austenitizing temperatures of 850, 900, and 950 °C, respectively. The corresponding residual stress on the surface of the specimen was −1031 ± 10, −1027 ± 6, and −1055 ± 9 MPa. These results indicate that prior cryogenic treatment does not significantly affect the amount of retained austenite and residual stress after the induction hardening treatment. The induction-hardened specimens were considered to be in a re-hardened state. Therefore, the benefits of cryogenic treatment are not obvious. The percentage of retained austenite and surface residual stress of the 12 kW induction-hardened SUJ3 specimens with various prior heat treatments are summarized in Table 7.

### 3.3. The Effect of Austenitizing Temperature, Cryogenic Treatment, and Induction Hardening Treatment on Dry Wear Behavior of the JIS SUJ3 Specimen

As demonstrated in the above sections, the effects of different austenitizing temperatures, cryogenic treatment, and induction hardening treatment were investigated. The microhardness, retained austenite content, and surface residual stress were measured and are summarized in Figure S5. Within the limitations of the present work, data on the depth of residual compressive stress and the changes in the specimen’s diameters after different heat treatments are not available and will be addressed elsewhere.

**Figure 13:**
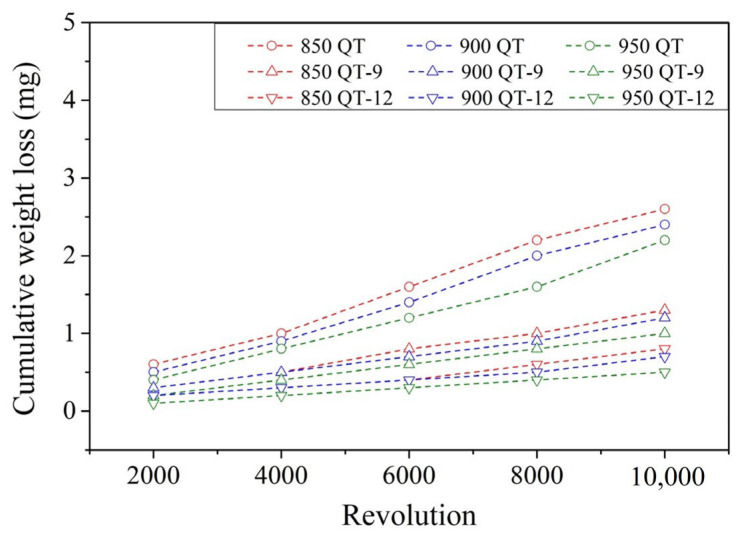
The cumulative weight loss profile of specimens without a prior cryogenic treatment.

**Figure 14:**
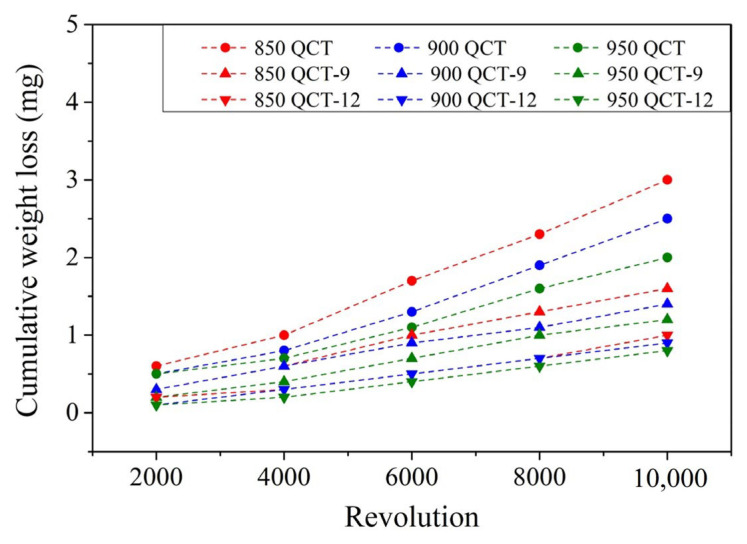
The cumulative weight loss profile of specimens with prior cryogenic treatment.

To investigate the dry wear behavior of specimens, a block-on-roller wear test with an applied contact load of 92 N and a roller rotation rate of 250 rpm was performed at room temperature. The cumulative weight losses of SUJ3 specimens with different prior austenitizing temperatures and induction hardening treatments with and without a prior cryogenic treatment are shown in Figure 13 and Figure 14, respectively. Cumulative weight loss increased with a prolonged revolution cycle. With induction hardening, the weight loss of the specimen significantly decreased. By increasing the power applied to the induction hardening treatment, weight loss can be further decreased. Similar behavior has been observed by Hu et al. [17] and Kaiser et al. [30]. It should be pointed out that the induction hardening treatment can induce significant compressive residual stress due to the rapid cooling rate. As a result, the wear resistance was improved.

**Figure 15:**
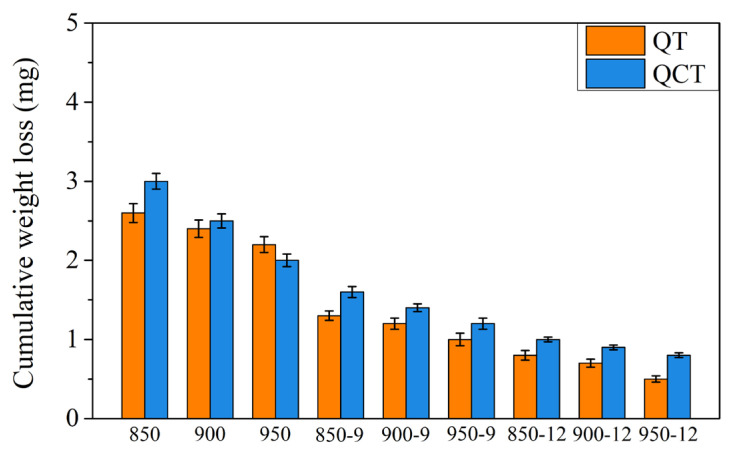
Comparison of weight loss among the specimens after the 10,000-cycle dry wear test.

Figure 15 shows the weight loss of the JIS SUJ3 specimen under different conditions after the 10,000-cycle dry wear test. As shown by the orange bars in Figure 15, the weight loss of heat-treated specimens without prior cryogenic treatment was 2.60 ± 0.11, 2.40 ± 0.10, and 2.20 ± 0.10 mg with austenitizing temperatures of 850, 900, and 950 °C, respectively. It can be decreased further with induction hardening. The weight loss of the 9 kW induction-hardened specimen without prior cryogenic treatment was 1.30 ± 0.09, 1.20 ± 0.05, and 1.00 ± 0.07 mg with an austenitizing temperature of 850, 900, and 950 °C, respectively. Then, it reduced to 0.80 ± 0.05, 0.70 ± 0.07, and 0.50 ± 0.03 mg at an induction power of 12 kW. It can be noted that the increase in austenitizing temperature and power applied for induction hardening treatment resulted in lower weight loss, which decreased after the dry wear test due to the increase in microhardness. However, specimens with prior cryogenic treatment do not exhibit a significant decrease in weight loss. It should be pointed out that the prior cryogenic treatment has lower compressive residual stress compared to those without a prior cryogenic treatment. Therefore, the wear resistance was not improved.

**Figure 16:**
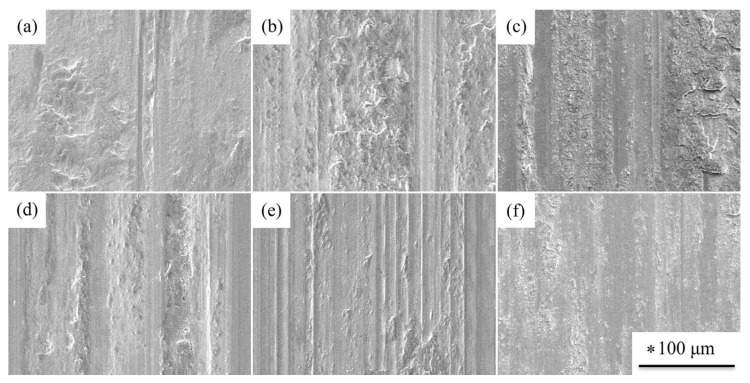
Surface morphology of worn specimens treated with the given conditions: (**a**) 850 QT, (**b**) 900 QT, (**c**) 950 QT, (**d**) 850 QCT, (**e**) 900 QCT, and (**f**) 950 QCT. * The scale bars were the same for these SEM images.

**Figure 17:**
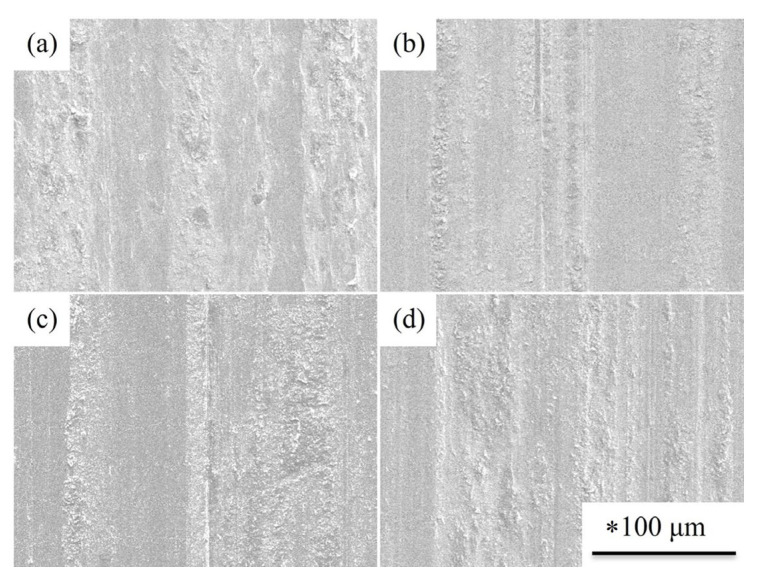
Surface morphology of worn specimens treated with the given conditions: (**a**) 950 QT-9, (**b**) 950 QCT-9, (**c**) 950 QT-12 and (**d**) 950 QCT-12. * The scale bars were the same for these SEM images.

The surface morphologies of the SUJ3 specimens with or without prior cryogenic treatment with different austenitizing temperatures are shown in Figure 16. The worn surface of the specimens was distributed by groove, scraped abrasive particles, and partial peeling. This includes abrasive wear and adhesive wear. Figure 17 shows the surface morphology of the induction-hardened SUJ3 specimens with and without prior cryogenic treatment with an austenitizing temperature of 950 °C. Compared to those without the induction hardening treatment (Figure 16c,f), it shows a smoother worn surface due to the lower weight loss.

## 4. Conclusions

In the present study, the JIS SUJ3 specimens underwent austenitizing treatment with different temperatures. Alternative cryogenic and induction hardening treatments were also investigated. The microhardness, surface residual stress, amount of retained austenite, and wear behaviors were investigated, and the following conclusions were drawn:

1. The residual tensile stress on the surface of the austenitized specimen was converted into compressive stress accompanied by cryogenic treatment, and a decrease in the amount of retained austenite can be observed.
2. By induction hardening, the microhardness and the residual compressive stress of austenitized and quenched specimens were significantly increased.
3. The prior austenitizing temperature and induction power can affect the induction hardening results. The induction hardening effect increased with increasing the prior austenitizing temperature and induction power. A prior austenitizing temperature of 950 °C and induction power of 12 kW produced optimal results. The maximum compressive residual stress reached −1083 MPa (on the surface), and the microhardness (in the induction-hardened subsurface region) was also the highest (920 ± 10 HV_0.3_).
4. The cryogenic treatment can decrease the amount of retained austenite. However, the induction-hardened specimens with a prior cryogenic treatment did not show a lower or even higher weight loss after the dry wear test. Thus, for bearing parts used in dry wear conditions, induction hardening the pre-austenitized specimen without prior cryogenic treatment was the optimal choice.

## Figures and Tables

**Table 1:** Chemical compositions (wt.%) of the JIS SUJ3 steel.

	C	Si	Mn	P	S	Cr	Ni	Mo	Cu	Fe.
JIS SUJ3 Spec.	0.95–1.10	0.40–0.70	0.90–1.15	≤0.025	≤0.025	0.90–1.20	≤0.25	≤0.08	≤0.30	Bal.
Specimens	1.05	0.326	1.02	0.0106	0.0051	1.14	0.0023	0.0014	0.0865	Bal.

**Table 2:** Prior heat treatment and induction hardening process parameters and code.

Austenitizing Temperature	Cryogenic Treatment	Induction Power (kW)	Code
850 °C	No	No	850 QT
900 °C			900 QT
950 °C			950 QT
850 °C	Yes	No	850 QCT
900 °C			900 QCT
950 °C			950 QCT
850 °C	No	9	850 QT-9
900 °C			900 QT-9
950 °C			950 QT-9
850 °C	Yes	9	850 QCT-9
900 °C			900 QCT-9
950 °C			950 QCT-9
850 °C	No	12	850 QT-12
900 °C			900 QT-12
950 °C			950 QT-12
850 °C	Yes	12	850 QCT-12
900 °C			900 QCT-12
950 °C			950 QCT-12

**Table 3:** The parameters of the X-ray diffractometer.

Diffractometer Parameters	Specification/Values
Target	Cr
Diffraction plane (h,k,l)	α Fe (211)
Bragg angle for diffraction (2θ)	156.5°
X-ray tube current	1.5 mA
X-ray tube voltage	30 kV
Irradiation time	15 s
Collimator diameter	2 mm
Collimator distance	51 mm

**Table 4:** The microhardness, the percentage of retained austenite and surface residual stress profile of SUJ3 specimen after austenitizing and quenching treatments.

Sample Code	Microhardness (HV_0.3_)	Retained Austenite (%)	Surface Residual Stress (MPa)
850 QT	715 ± 5	3.4 ± 0.3	53 ± 12
900 QT	725 ± 8	10.4 ± 0.7	78 ± 17
950 QT	728 ± 15	15.5 ± 0.6	115 ± 15

**Table 5:** The microhardness, the percentage of retained austenite, and the surface residual stress profile of the SUJ3 specimen at given conditions.

Sample Code	Microhardness (HV_0.3_)	Retained Austenite (%)	Surface Residual Stress (MPa)
850 QCT	718 ± 3	3.5 ± 0.2	−61 ± 11
900 QCT	723 ± 5	5.2 ± 0.3	−92 ± 8
950 QCT	730 ± 8	7.4 ± 0.3	−132 ± 14

**Table 6:** The percentage of retained austenite and surface residual stress of 9 kW induction-hardened SUJ3 specimens.

Sample Code	Retained Austenite (%)	Surface Residual Stress (MPa)
850 QT-9	5.3 ± 0.7	−951 ± 13
850 QCT-9	4.9 ± 0.4	−900 ± 20
900 QT-9	7.3 ± 0.5	−977 ± 17
900 QCT-9	6.5 ± 0.6	−922 ± 15
950 QT-9	9.4 ± 0.9	−974 ± 9
950 QCT-9	8.7 ± 0.7	−915 ± 19

**Table 7:** The percentage of retained austenite and surface residual stress of 12 kW induction-hardened SUJ3 specimens.

Sample Code	Retained Austenite (%)	Surface Residual Stress (MPa)
850 QT-12	6.2 ± 0.3	−1068 ± 3
850 QCT-12	5.8 ± 0.2	−1031 ± 10
900 QT-12	8.9 ± 0.4	−1073 ± 11
900 QCT-12	8.5 ± 0.4	−1027 ± 6
950 QT-12	11.8 ± 0.3	−1083 ± 5
950 QCT-12	11.3 ± 0.4	−1055 ± 9

## Data Availability

The original contributions presented in this study are included in the article. Further inquiries can be directed to the corresponding author.

## Supplementary Materials

The following supporting information can be downloaded at https://www.mdpi.com/article/10.3390/ma18081797/s1. Figure S1: Temperature time diagrams for (a) QT and (b) QCT treatments. Figure S2: (a) Schematic diagram of abrasion tester (block-on-roller type); (b) experimental setup of the block (specimen) and roller on the abrasion tester. Figure S3: Microstructure images with a higher magnification of the (a) as-received SUJ3 specimen, (b) 850 QT, (c) 900 QT, and (d) 950 QT specimens. Figure S4: Microstructure images with a higher magnification of (a) 850 QCT (b) 900 QCT, and (c) 950 QCT specimens. Figure S5: (a) The microhardness (the original HV before heat treatment was shown as the red line in the figure), (b) the amount of retained austenite, and (c) the surface residual stress profile of samples after different heat treatment methods. Table S1. Powder diffraction file of martensitic phase (PDF No. 44-1290). Table S2. Powder diffraction file of austenite (PDF No. 65-4150).
