# Supplementary material for: Effects of Prior Heat Treatment and Induction Hardening on the Properties of JIS SUJ3 Bearing Steel
Source: Materials (Basel). 2025 Apr 15;18(8):1797. doi: 10.3390/ma18081797 (PMC12029054; doi:10.3390/ma18081797)
Supplement: Supplementary file 1 [file materials-18-01797-s001.zip › materials-3528263-supplementary.pdf]

Figure S1(a) and S1(b) show the temperature-time diagrams for QT and QCT treatments used in the present study, respectively.

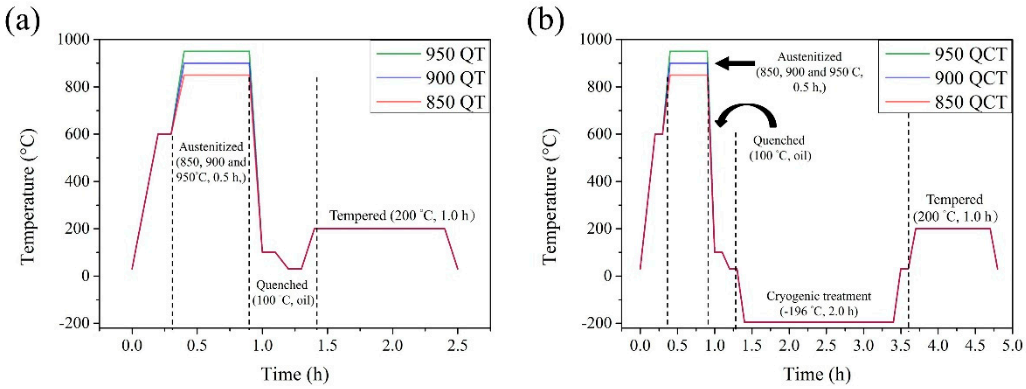

Figure S1. Temperature time diagrams for (**a**) QT and (**b**) QCT treatments.

Dry wear tests were performed using a multi-functional abrasion tester (PLINT TE53, Phoenix Tribology Ltd., Kingsclere, UK). A schematic illustration of the experimental setup is shown in Fig. S2a. The abrasion tester is a block-on-roller contact instrument in which a cubic block (specimen) is in contact with a roller as shown in Fig. S2b. The block (specimen) experiences one contact cycle per revolution at a constant contact normal load.

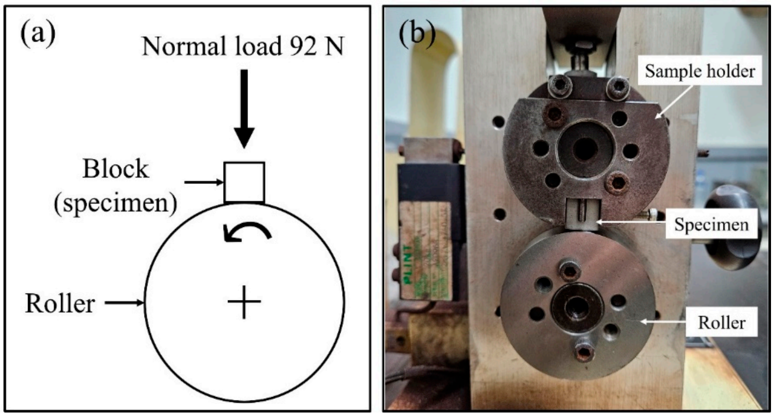

Figure S2. (**a**) schematic diagram of abrasion tester (block-on-roller type); (**b**) experimental setup of the block (specimen) and roller on the abrasion tester.

Figure S3 shows the microstructure (carbide, martensite and retained austenite) of the austenitized and quenched JIS SUJ3 specimens. The proportion of the cementite decreased with the increasing austenitization temperature. However, the higher austenitization temperature will increase the amount of retained austenite due to the decrease in Ms temperature.

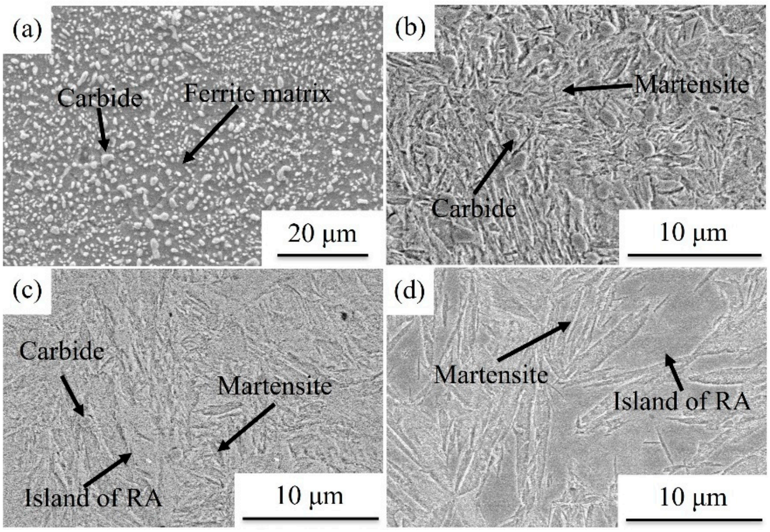

Figure S3. Microstructure images with a higher magnification of the (**a**) as-received SUJ3 specimen, (**b**) 850 QT, (**c**) 900 QT, and (**d**) 950 QT specimens.

Figure S4 shows the microstructure of the austenitized and quenched JIS SUJ3 specimens after cryogenic treatment. After cryogenic treatment, the amount of the retained austenite was reduced due to the heat exchange.

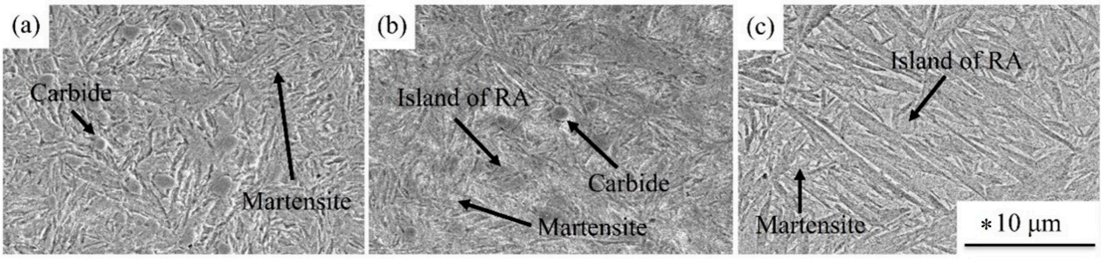

Figure S4. Microstructure images with a higher magnification of (**a**) 850 QCT (**b**) 900 QCT, and (**c**) 950 QCT specimens. * The scale bars were the same for these SEM images.

The microhardness, the amount of retained austenite and residual stress of different heat treatment methods were summarized in Figure S5 (a), (b) and (c), respectively. Figure S5 (a) shows that the microhardness increased significantly at the subsurface region of specimens after induction hardening treatment (increase from 725 $HV_{0.3}$ up to 920 $HV_{0.3}$). In comparison, the prior cryogenic treatment cannot significantly increase or decrease the microhardness in the subsurface region of the induction-hardened specimens. Figures S5 (b) and (c) show the retained austenite and surface residual stress profile of the samples after different heat treatment methods. The prior cryogenic treatment can significantly reduce the amount of retained austenite (decrease by less than 8%) and change the surface residual stress to a compressive state (from a tensile state) in the austenitized and quenched specimens. However, the prior cryogenic treatment can't significantly affect the amount of retained austenite and surface residual stress in induction-hardened specimens.

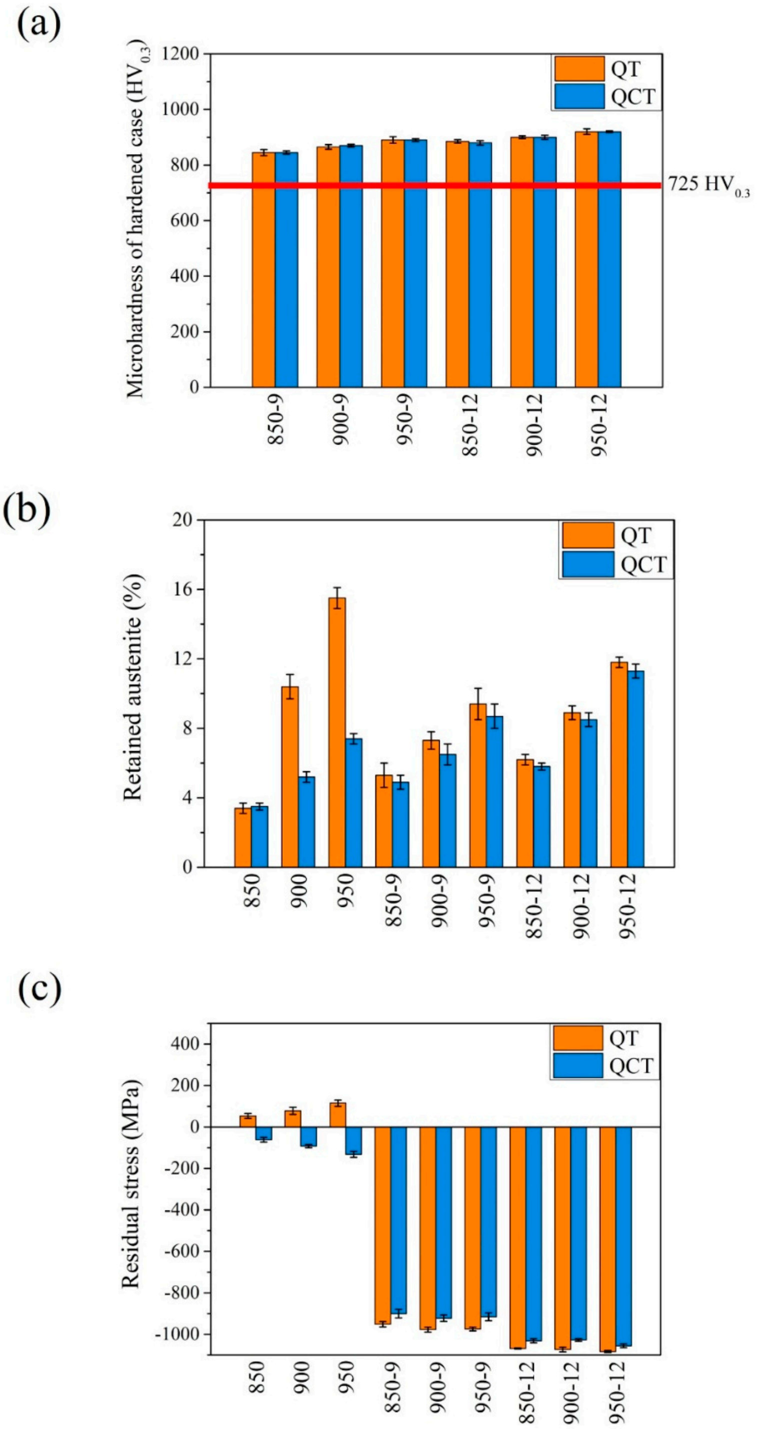

Figure S5. (**a**) the microhardness (the original HV before heat treatment was shown as the red line in the figure), (**b**) the amount of retained austenite, and (**c**) the surface residual stress profile of samples after different heat treatment methods.

Tables S1 and S2 are the powder diffraction files (PDF) for the martensitic phase and austenite.

Table S1. Powder diffraction file of martensitic phase (PDF No. 44-1290).

Martensite 44-1290		
2θ	(h,k,l)	I (%)
44.220	(101)	100
44.841	(110)	50
63.342	(002)	10
65.281	(200)	17
80.921	(112)	19
82.245	(211)	35
97.650	(202)	12
99.416	(220)	6
112.68	(103)	11
116.53	(301)	11
117.01	(310)	16
135.61	(222)	10
165.30	(213)	26

Table S2. Powder diffraction file of austenite (PDF No. 65-4150).

Austenite 65-4150		
2θ	(h,k,l)	I (%)
42.946	(111)	100
50.009	(200)	51
73.421	(220)	31
89.007	(311)	34
94.128	(222)	10
115.42	(400)	5
134.21	(331)	18
141.87	(420)	18
